# Preregistration of Preclinical Animal Studies as a Tool for Refinement: A Feasibility Study Comparing the German Animal Study Registry and Preclinicaltrials.eu

**DOI:** 10.3390/ani16142226

**Published:** 2026-07-18

**Authors:** Lena Kistermann, Maja Strunk, Ivonne Jeanette Knorr, Steven R. Talbot, Lisa Ernst, René H. Tolba

**Affiliations:** 1 Institute for Laboratory Animal Science and Experimental Surgery, RWTH Aachen University, 52074 Aachen, Germany; mstrunk@ukaachen.de (M.S.); iknorr@ukaachen.de (I.J.K.); lernst@ukaachen.de (L.E.); rtolba@ukaachen.de (R.H.T.); 2 3R-Competence Network North Rhine Westfalia, Faculty of Medicine, RWTH Aachen University, 52074 Aachen, Germany; 3 Institute for Laboratory Animal Science, Hannover Medical School, 30625 Hannover, Germany; stalbot@ukaachen.de; 4 Institute of Medical Statistics, RWTH Aachen University, 52062 Aachen, Germany

**Keywords:** preregistration, preclinical animal research, reproducibility, refinement, reduction, 3R, laboratory animal science, open science

## Abstract

Preregistration of animal studies is increasingly promoted as a methodological Refinement that can reduce bias, prevent unnecessary duplication and ultimately benefit animal welfare. Despite dedicated open registries (i.e., the Animal Study Registry of the German Centre for the Protection of Laboratory Animals (ASR) and preclinicaltrials.eu (PCT)), uptake remains low. The perceived time burden and limited awareness are the most frequently mentioned barriers, although these registries have been available since 2018 and 2019. In this feasibility study, eleven researchers (Beginners *n* = 3, Trainees *n* = 3, Experts *n* = 5) preregistered two preclinical protocols (rat and pig) on each platform, while completion time, mouse clicks, cursor distance, and scroll events were registered. An anonymous survey collected free-text comments. Completion time was substantially longer on ASR than on PCT for both the rat protocol (99.5 ± 29.1 vs. 39.5 ± 4.9 min) and the pig protocol (53.1 ± 17.2 vs. 25.8 ± 8.5 min), with the same direction for the other parameters. All eleven participants preferred PCT, and completion time decreased with practice. Preregistration is technically feasible on either platform. However, structured training and targeted usability improvements could reduce the activation cost and support its role as a Refinement strategy in laboratory animal science.

## Simple Summary

Thorough planning and transparent reporting make animal studies more robust and reproducible, which helps avoid unnecessary duplication and makes the best use of every animal. Preregistration (i.e., publishing a study plan before data are collected) supports these goals and the principles of Replacement, Reduction and Refinement (the 3R). Two open registries are dedicated to preclinical animal research: the Animal Study Registry in Germany (ASR) and preclinicaltrials.eu (PCT) in the Netherlands. Uptake on both remains low, with researchers most often citing the added time and effort and a general lack of awareness. We asked eleven users with differing levels of regulatory experience to preregister real preclinical protocols on both platforms while recording the time required and their mouse and scrolling activity. Participants became substantially faster as the session went on, regardless of their prior experience. Across all workflow metrics, the European platform took less time and fewer navigational actions than the German platform, and all eleven participants preferred it. Even brief practical training and targeted usability improvements could lower the perceived burden of preregistration and, in turn, benefit the welfare of laboratory animals.

## 1. Introduction

Reproducibility is a recognised challenge in preclinical animal research [1,2,3]. Several distinct factors contribute. Incomplete reporting of methods hinders independent replication difficult and selective publication of positive results obscures the full evidence base. Post hoc adaptation of analyses (i.e., p-hacking and as hypothesising after results are known (HARKing)) inflates the apparent strength of effects and negative or null findings, although equally informative, are less likely to be published [4,5]. These consequences extend beyond translational research. In basic science, the accumulated evidence from animal studies is systematically skewed toward positive findings, while results that could inform future designs remain unpublished. The implications are also ethical: an unreliable literature is a weak foundation for follow-up and translational decisions [6,7], and unrecognised duplication of unpublished work means more animals may be used than necessary to establish what would otherwise be a single robust result.

Preregistration (i.e., the prospective registration of a study protocol in a public registry before data collection begins) has been proposed as one structural response to these problems [4,8]. By documenting the research questions, hypotheses, sample size, primary and secondary outcomes, methods and analysis plan in advance, it creates a verifiable separation between confirmatory and exploratory analyses and reduces the scope for HARKing, selective outcome reporting and post hoc reframing [4]. The principle is well established in clinical research: after it became clear that trials with negative results frequently went unpublished and thereby distorted the apparent efficacy of medical interventions, prospective trial registration was made a precondition for publication. ClinicalTrials.gov, launched in 2000, now lists more than 530,000 registered studies from over two hundred countries [9]. The same logic is increasingly applied to animal studies. The Animal Research: Reporting of In Vivo Experiments (ARRIVE) 2.0 guideline [10] and the Planning Research and Experimental Procedures on Animals: Recommendations for Excellence (PREPARE) guideline [11] both endorse prospective registration. Together, these three elements are complementary: PREPARE structures the planning, preregistration generates a public, dated and citable record, and ARRIVE specifies reporting. Beyond transparency, preregistration is increasingly framed as a contribution to the 3Rs (i.e., Replacement, Reduction and Refinement) of animal research [12,13]. The link to Refinement and Reduction operates through study planning. A registry entry obliges the researcher to specify the hypothesis, primary endpoint, sample-size justification and analysis plan before any animal is used, making studies less prone to underpowered designs and post hoc cohort extension [13,14,15]. A public, searchable record of planned experiments also helps detect and avoid unnecessary duplication of completed but unpublished work [5,8]. Preregistration is thus not only an integrity tool but also an animal-welfare tool.

Three open registries with international scope accept animal study preregistrations. The Open Science Framework (Center for Open Science, Charlottesville, VA, USA) registry is a general-purpose research registry that accommodates, but is not restricted to, animal studies. Two platforms are dedicated to preclinical animal research: the Animal Study Registry (ASR), launched in January 2019 by the German Centre for the Protection of Laboratory Animals (Bf3R) at the German Federal Institute for Risk Assessment (BfR) [14] and preclinicaltrials.eu (PCT), launched in 2018 by University Medical Center Utrecht and the Netherlands Heart Institute [8,16]. A 2022 declaration of common standards by representatives of all three platforms describes their convergence on a shared set of minimum reporting fields [17]. Both ASR and PCT are free to use, assign a citable Digital Object Identifier (DOI) to each registration and offer an embargo function (up to five years on ASR; one year by default on PCT, extendable) to protect intellectual property until publication (see Table 1). Because the present study focuses on animal-specific registries designed around the preclinical workflow, the empirical comparison is restricted to ASR and PCT.

Despite their availability, uptake of either animal-specific registry has remained relatively low [14,16]. As of 21 May 2026, 265 studies were registered at ASR, and 262 were registered on PCT, excluding studies under embargo.

Among the explanations frequently given are the perceived additional time burden and limited awareness of these registries [13,17,18,19]. To our knowledge, however, the time and effort actually required to complete a registration have not been quantified empirically, nor has it been examined whether prior regulatory experience confers an advantage or whether the burden diminishes with practice.

In the present study, we recorded workflow data while eleven researchers, with varying levels of experience in laboratory animal science, preregistered two real preclinical study protocols (one rat protocol, one pig protocol) on each platform. We measured completion time, mouse clicks, cursor travel distance and scroll events. The research questions to be addressed were as follows:

- Does task performance improve when users complete multiple preregistration tasks in sequence, and is the improvement consistent across the four outcome measures?
- Is the level of prior experience (Beginner, Trainee, Expert) indicative of baseline performance?
- Do the two platforms differ in terms of workflow efficiency and user-perceived usability?
- What qualitative usability themes emerge from a parallel anonymous user-experience survey of both platforms?

## 2. Materials and Methods

A within-subjects feasibility study was conducted, in which each participant completed four preregistration tasks. The four tasks resulted from crossing two registries (ASR and PCT) with two real preclinical animal study protocols: one rat protocol and one pig protocol. Experience group (Beginner, Trainee, Expert) served as the between-subjects factor and trial position within the schedule (1–4; 1 ASR rat protocol, 2 PCT rat protocol, 3 ASR pig protocol, 4 PCT pig protocol) served as the within-subjects factor. All participants gave written informed consent before their first session, were free to withdraw from the study at any time without any disadvantage and received no monetary compensation. The study protocol received a positive vote from the Ethics Committee of the Medical Faculty of RWTH Aachen University (case number EK 24-258).

Eleven participants were recruited from the staff of the Institute for Laboratory Animal Science and Experimental Surgery (RWTH Aachen, Aachen, Germany) and assigned to one of three experience groups based on their prior experience with animal study application procedures, in conjunction with their concurrent professional experience in laboratory animal science. Beginners (*n* = 3) had no prior experience with regulatory animal study applications and a median of approximately one year of professional experience. Trainees (*n* = 3) had supervised experience with regulatory applications and a median of approximately three years of experience. Experts (*n* = 5) had regular and independent experience with regulatory applications and a median of more than ten years of experience, with the most experienced participant exceeding twenty years. By occupation: Beginners were a veterinarian, an administrative veterinary officer and a trainee veterinary assistant. Trainees were a veterinarian and two PhD candidates. Experts were an experienced MD, a PhD, a research technician, an animal care specialist, a state-certified engineer in biology and a veterinarian. All participants had at least a basic working knowledge of Microsoft Office 365 (Word, Excel and PowerPoint), which they used in their daily work.

**Figure 1:**
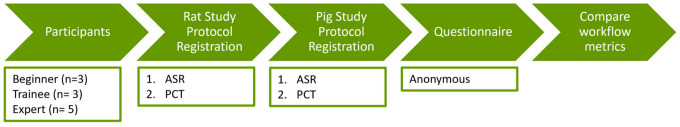
Study design and task sequence.

The task order was fixed, not counterbalanced. Ten of the eleven participants followed the sequence first ASR/Rat, second PCT/Rat, third ASR/Pig, fourth PCT/Pig (see Figure 1). One participant (Expert) deviated by completing PCT/Pig before ASR/Pig at trial positions 3 and 4. The study was planned as a descriptive feasibility and usability evaluation using a convenience sample of the institute’s staff. Accordingly, no formal a priori sample-size calculation was performed. Workflow metrics are summarised descriptively (mean ± standard deviation), and the direction of differences is reported across all comparisons.

Because ASR was administered before PCT for both species in ten of the eleven participants, registry and species are almost fully confounded with trial position. The inferential analysis, therefore, modelled trial position, and no inferential test of the ASR–PCT difference was performed. The platform metrics remain reported descriptively. Each of the four outcome measures was analysed with a linear mixed-effects model with trial position (continuous, 1–4), experience group and their interaction as fixed effects and a random intercept per participant. Random slopes for trial position could not be estimated at this sample size, so random-intercept-only models were used, which is acknowledged as a limitation. Type III F-tests with Kenward–Roger-corrected denominator degrees of freedom were used, the significance level was α = 0.05 (two-sided), and *p*-values were Holm-corrected within each fixed effect across the four outcomes. Effect sizes are reported as approximate partial η^2^. Two sensitivity analyses were performed: adding a quadratic term for trial position and refitting on log-transformed outcomes. Analyses were conducted in R 4.4.1 with the lme4 (2.0-1), lmerTest (3.2-1), emmeans (2.0.3), effectsize (1.0.3) and pbkrtest (0.5-5) packages.Prespecified confirmatory analyses remain reserved for a planned counterbalanced follow-up study.

Completion time, defined as the elapsed time from starting the registration to reaching the final review step of the protocol, was the primary outcome and was recorded in minutes. Three secondary outcomes capturing navigation effort were derived from input-event logs, using OdoPlus (version 16.281): total mouse clicks, cumulative cursor travel distance (in metres) and number of scroll events.

After the data-collection period, participants were invited to complete an anonymous online questionnaire with two free-text fields per platform on technical difficulties and desired improvements. Anonymity precluded linkage of questionnaire responses to individual workflow records.

The free-text responses were analysed by inductive (conventional) content analysis [20]. Two dimensions (i.e., technical difficulties during registration and suggested improvements) were coded separately into categories derived directly from the responses and are reported in the two sections of Table 2. Each category was counted once per participant and platform (at most one entry per participant, platform and category), and entries were summed per platform. The questionnaire was exploratory and hypothesis-generating, intended to surface candidate usability issues for a subsequent confirmatory study rather than to form a stand-alone qualitative dataset. Given the small volume of free text, whose comments pointed in a similar direction, coding used a simple set of counting rules applied by one author and checked by a second, without a formal inter-coder reliability statistic. The resulting counts are reported as descriptive, hypothesis-generating context rather than as directly comparable to the quantitative workflow metrics.

## 3. Results

### 3.1. Within-Session Performance Trajectory and Prior Experience

Across all eleven participants, performance improved substantially over the four tasks. Mean completion time fell from 99.5 ± 29.1 min at the first trial position to 24.3 ± 5.4 min at the fourth. The linear effect of trial position was significant for completion time (F(1,30) = 45.16, *p* < 0.001, partial η^2^ = 0.601; an estimated 21.3 min less per task) and for total clicks (F(1,30) = 40.19, *p* < 0.001, partial η^2^ = 0.573), cursor distance (F(1,29) = 31.95, *p* < 0.001, partial η^2^ = 0.524) and scroll events (F(1,30) = 17.14, *p* < 0.001, partial η^2^ = 0.364). All four effects remained significant after Holm correction. A significant quadratic term for completion time, total clicks and cursor distance (all *p* < 0.05) indicated diminishing returns, and log-transformed models reproduced the same pattern, confirming robustness to the right-skewed distributions. Because trial position is almost fully confounded with registry, this trajectory captures the combined effect of practice and platform across the session.

Prior regulatory experience did not predict baseline performance on any of the four outcomes (all *p* = 1.0 after Holm correction), and the interaction between trial position and experience group was not significant for any outcome (all *p* > 0.50), indicating comparable rates of improvement across groups. Descriptively, Beginners tended to take longer than Experts at the first trial position.

### 3.2. Platform-Specific Comparison

**Figure 2:**
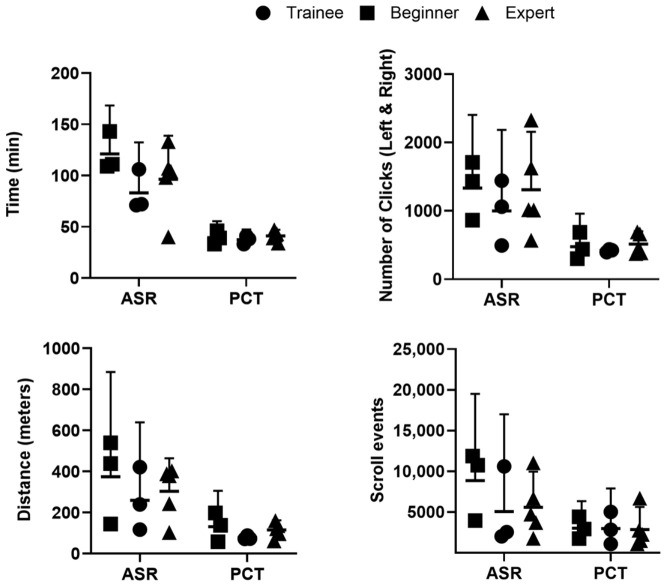
Workflow parameters for the rat study protocol.

**Figure 3:**
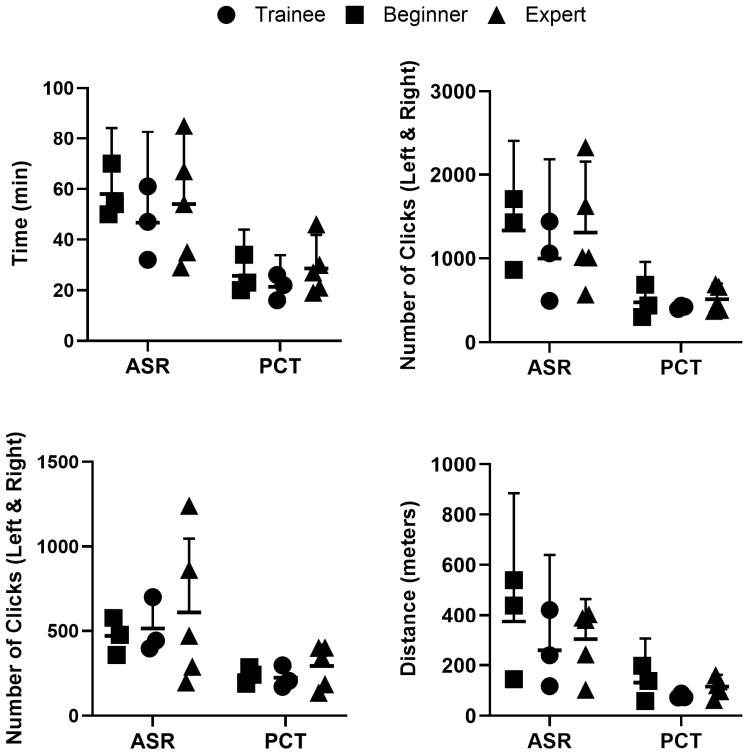
Workflow parameters for the pig study protocol.

Within each protocol, the recorded values differed in the same direction between the two platforms for all four workflow metrics (Figure 2 and Figure 3).

In terms of completion time, the preregistration on ASR took longer than PCT in both protocols: 99.5 ± 29.1 min vs. 39.5 ± 4.9 min for the rat protocol (a difference of ~60 ± 8.8 min) and 53.1 ± 17.2 min vs. 25.8 ± 8.5 min for the pig protocol (~27 ± 6.4 min).

The three navigation metrics showed the same direction. Mouse clicks were higher on ASR than PCT (rat: 1231 ± 539 vs. 477 ± 137; pig: 547 ± 296 vs. 260 ± 93), as were cursor distances (rat: 310.8 ± 147.8 m vs. 109.4 ± 45.2 m; pig: 145.1 ± 93.4 m vs. 69.9 ± 36.8 m) and scroll events (rat: 6341 ± 3991 vs. 2945 ± 1776; pig: 4604 ± 1979 vs. 1784 ± 907).

The direction was the same across all metrics and both protocols (Figure 2 and Figure 3): in all eight comparisons, ASR values were higher on both protocols (Figure 2 and Figure 3), with ASR values 2.0–2.8 times the corresponding PCT values.

### 3.3. User Platform Preference

At the conclusion of all sessions, all eleven participants expressed a preference for PCT over ASR. This unanimous preference (11 of 11 participants, 100%) is in line with the quantitative workflow data and with the qualitative comments and is notable given the diversity of experience levels (Beginners, Trainees and Experts).

### 3.4. User-Experience Survey: Free-Text Comments

The survey was completed by ten respondents, all of whom had also completed the workflow tasks (Table 2). On ASR, the most frequent difficulty was inserting figures and graphics (7 of 10), as protocol diagrams and tabular sample-size justifications could only be uploaded as separate files. System errors were raised by six respondents (e.g., the editor could freeze or lose input on text entries > 500 characters), and six reported input-field usability issues, notably over-granular fields for related items better entered as a single block. Copy-and-paste, registration/login, and formatting issues were each raised by four respondents. Comments on PCT were fewer and narrower in scope, with no respondent reporting system errors, saving issues, error messages or loading problems. Desired improvements reflected the difficulties, with easier image insertion being the most-named ASR improvement (7 of 10).

## 4. Discussion

A first-time user who needs approximately 100 min for an initial preregistration can be expected to complete subsequent registrations faster, and this improvement was in the same direction across all four outcome measures. The four workflow parameters capture complementary aspects of effort: completion time reflects overall task duration, mouse clicks the number of discrete interaction steps, cursor distance the spatial navigation imposed by the interface layout and scroll events the page length and content density that must be traversed. Therefore, the often-cited time effort of preregistration is not a permanent additional burden but largely an activation cost that falls steeply with experience. Day-to-day work in the laboratory is tightly scheduled, and the regulatory load is rising, yet our data indicate that the activation cost is real but neither as large nor as persistent as is sometimes assumed and that institutional support structures could absorb a meaningful share of it [18,19].

Prior experience with regulatory animal study applications did not predict baseline workflow performance in any of the four outcome measures. Descriptively, Beginners required somewhat longer than Experts at the first trial position, but the pattern was not monotonic, and Trainees were, on average, the fastest. This null result is informative: it suggests that the activation cost is primarily structural, a function of the interface and the unfamiliarity of the task, rather than of individual skill. In line with this, the participants’ shared daily use of Microsoft Office 365 suggests a comparable baseline of data-entry competence across the sample. Taken together, these observations indicate that targeted training and usability improvements would benefit researchers across the full experience range, not only newcomers.

The platform comparison drew on three distinct sources: workflow metrics, unanimous user preference and qualitative themes. Across all workflow metrics, ASR required more time and navigational effort than PCT. The mean difference (~27 ± 6.4 min for the pig protocol; ~60 ± 8.8 min for the rat protocol; 755 additional clicks for the rat protocol; 287 additional clicks for the pig protocol) is substantial in practical terms. Because ten of the eleven participants completed ASR before PCT, platform and practice position remain confounded, so the observed differences cannot be attributed to platform properties, but rather to task practice within the present design. Descriptively, the difference was in the same direction across completion time and the three navigation metrics, with ASR values 2.0–2.8 times the corresponding PCT values. Alongside the workflow metrics, all eleven participants preferred PCT, and their stated reasons pointed to specific, addressable interface problems on ASR (figure insertion, instability under longer text input, over-granular fields and copy-and-paste failures).

A planned follow-up study will address these limitations through a fully counterbalanced crossover design, a larger multi-institutional sample and prespecified confirmatory analyses of the platform difference. This is a longer-term effort whose results are not expected soon, so the present descriptive findings stand on their own.

Many items required for a registry entry overlap with information researchers already prepare for ARRIVE 2.0 reporting [10], the PREPARE planning checklist [11] and national approval under Directive 2010/63/EU [21], so institutions with a PREPARE-aligned workflow are well placed to lower the entry hurdle further [4,8,14,17]. The platforms differ practically in their embargo policy: ASR allows up to five years by default, whereas PCT applies a one-year embargo, extendable on request.

### Strengths and Limitations of the Study

The study has several strengths:

- Real preclinical protocols and real users on the live registry interfaces (not mock-ups) make the workflow figures directly informative for routine adoption.
- Alongside the metrics, a free-text survey added a qualitative layer whose findings are in line with the quantitative results.
- A unanimous, cross-experience preference for PCT (11 of 11; 100%) was expressed after participants had completed the workflow on both platforms.
- Platform differences were in the same direction across all metrics, favouring PCT.
- Both platforms confirmed no major interface or database changes during data collection.

We are also aware of several limitations:

- No counterbalancing: Ten of eleven participants began with ASR before PCT, so differences cannot be attributed exclusively to platform properties. The one deviating participant is insufficient to offset this, and causal conclusions would require a fully counterbalanced crossover design.
- Single-institution German sample, so context-specific factors (e.g., Directive 2010/63/EU familiarity, German-language infrastructure) may limit generalisability.
- As a descriptive feasibility study with one institutional sample, no a priori sample-size calculation was performed. The mixed-effects models used a random intercept per participant. Random slopes could not be estimated at this sample size, so the trajectory estimates should be read as approximate.

Together, these strengths as well as limitations frame the present findings as descriptive and hypothesis-generating. The agreement across workflow metrics, user preference and qualitative themes gives the observed patterns practical relevance, while the fixed task order means any platform difference remains a descriptive observation rather than a causal effect.

## 5. Conclusions

Preregistration of preclinical animal studies on ASR and PCT is technically feasible across all experience levels, and the time required falls steeply with practice. The activation cost on which researchers and institutions tend to focus is real but largely a one-off investment rather than a permanent additional burden. Both platforms allowed participants to complete the workflow. Descriptively, the observed differences were in the same direction across the workflow metrics, the unanimous user preference (11 of 11 participants) and the qualitative survey themes: irrespective of prior regulatory experience, recorded time and navigational effort were lower for PCT than for ASR, and every participant preferred PCT. Because platform and practice position were confounded in the non-counterbalanced design, the extent to which these differences reflect platform properties rather than practice cannot be established from the present descriptive data. The specific, addressable usability issues identified, together with brief institutional training, could meaningfully lower the entry hurdle for first-time users, helping to establish preregistration as a routine Refinement strategy in preclinical animal research.

## Figures and Tables

**Table 1:** Key features of the two open animal study registries compared in this study.

Feature	Animal Study Registry (ASR)	Preclinicaltrials.eu (PCT)
Operator	Bf3R/BfR (Germany)	UMC Utrecht and Netherlands Heart Institute
Launch	January 2019	2018
Scope	Preclinical animal studies	Preclinical animal studies
Cost	Free	Free
Languages	German, English	English
DOI assigned	Yes	Yes
Embargo	Up to five years	Default one-year, extendable
Suitable study types	Both confirmatory and exploratory; any animal species; in vivo and ex vivo	Both confirmatory and exploratory; any animal species; in vivo and ex vivo
Pilot/main-study linkage	Not yet supported within a single record	Not yet supported within a single record

Side-by-side comparison of the two open registries that currently accept and are suitable for animal study preregistrations.

**Table 2:** Reported technical difficulties and suggested usability improvements on each platform.

**Technical Difficulties**	**ASR**	**PCT**
System crashes	6	0
Copy-and-paste issues (incl. character errors)	4	1
Registration/login issues	4	1
Formatting issues (tables, bullet points, numbering, special characters)	4	3
Saving issues/data loss	2	0
General error messages	1	0
Problems inserting figures/graphics	7	3
Loading/performing issues	1	0
Navigation/scrolling issues	2	2
Input usability issues (resizing, minimising, cursor reset)	6	1
Total	37	11
**Suggested Improvement**	**ASR**	**PCT**
Improved formatting (tables, bullet points, text formatting)	4	2
Easier image insertion	7	1
Fix the copy-and-paste functionality	3	0
Better guidance/clearer field instructions	4	1
Warnings and autosave guidance	1	0
Restructure/split fields (reduce fragmentation or overly large fields)	4	2
Allow multiple selections (e.g., study stage/design)	2	0
Separate pilot and main study registration	2	2
Integration of score sheets	1	1
Increase error tolerance/reduce system rigidity	1	0
User-interface/navigation improvements	2	0
Language guidance/accessibility	1	1
Total	32	10

Total number of qualitative themes from the free-text field regarding the technical difficulties and recommended improvements of the user-experience questionnaire (*n*= 10). Numbers are descriptive context, not statistical comparisons.

## Data Availability

The raw data supporting the conclusions of this article will be made available by the authors on reasonable request.

## Abbreviations

The following abbreviations are used in this manuscript:

3R	Replacement, Reduction, Refinement
ARRIVE	Animal Research: Reporting of In Vivo Experiments
ASR	Animal Study Registry
Bf3R	German Centre for the Protection of Laboratory Animals
BfR	German Federal Institute for Risk Assessment
DOI	Digital Object Identifier
PCT	preclinicaltrials.eu
PREPARE	Planning Research and Experimental Procedures on Animals: Recommendations for Excellence
